# Dr. Edward N. Brush’s Anniversary Dinner

**Published:** 1909-02

**Authors:** 


BUFFALO MEDICAL JOURNAL.

**A Monthly Review of Medicine and Surgery.**

*EDITOR:*

WILLIAM WARREN POTTER, M. D.

All communications, whether of a literary or business nature, books for reviewand exchanges, should be addressed to the editor, 238 DELAWARE AVENUE, BUFFALO, N. Y.

VOL. LXIV. FEBRUARY, 1909. No. 7

### Dr. Edward N. Brush's Anniversary Dinner.

MANY friends of Dr. Edward N. Brush, of the Sheppard and Enoch Pratt Hospital of Baltimore, wishing to express their personal regard for him and their appreciation of the great service he has rendered to the public and private care of the insane, recently tendered him a public dinner upon his completion of thirty years of service in the institutions of New York, Pennsylvania and Maryland. The committee of arrangements consisted of G. Alder Blumer, Providence, R. I.; W. A. Paton, Lakewood, N. J.; John B. Chapin, Philadelphia; and Hugh H. Young, Charles M. Franklin, and Henry M. Hurd of Baltimore.

The banquet was served Friday evening, December 11, 1908, in the handsomely furnished dining room of the Baltimore Club. The individual tables were adorned with carnations and the menu was quite elaborate, though the price was appropriately fixed at the modest sum of $5.00 a plate.

Dr. G. Alder Blumer, of Providence, R. I., presided and introduced the speakers, who were Attorney-General Charles J. Bonaparte, Dr. Ira Remsen, president of the Johns Hopkins University; Dr. George A. Pope, Dr. C. K. Mills, of Philadelphia, and Dr. William A. White, of Washington, head of the Government Hospital for the Insane. A cablegram from Dr. Osler at Paris was read which said: "Greeting to Brush; only gratitude and affection can pay the debt of the profession to him."

The guests, some seventy in number, were nearly all physicians, most of whom were distinguished psychiatrists and the few laymen present were connected more or less directly with the administration of hospitals for the treatment of the insane.

The *Maryland Medical Journal* in its issue for December, 1908, gives an account of the Sheppard and Enoch Pratt Hospital, located at Towson, near Baltimore, of which Dr. Brush is Medical

Superintendent. Incidentally it contains a sketch of Dr. Brush from which we extract liberally.

With their customary circumspection, the trustees sought for medical superintendent the best-equipped alienist to be obtained. Dr. Edward N. Brush, assistant physician at the Pennsylvania Hospital for the Insane, Philadelphia, was selected. A graduate of the University of Buffalo, and at one time lecturer there on electro-therapeutics, Dr. Brush had, after four years of general hospital work and private practice, become interested in psychiatry, and had pursued his studies along this line for nearly seven years as assistant physician in the State Hospital for the Insane at Utica, and then for seven years more had held the same position of responsibility in the Pennsylvania Hospital. He was called in his 39th year, in the full maturity of his powers, to the new work at Sheppard, of which, by the wise determination of the trustees, he was given absolute medical control. Dr. Brush's annual reports show that he at once recognised the opportunity to create at Sheppard a hospital not only in name, but in fact, an opportunity of which the fullest advantage has been taken. In eulogy of one who is still an active participant in medical progress among us we must of necessity be very reticent. A detailed account of his views concerning the treatment of the insane is given in the chapter on hospital care of the insane which he has contributed to Hare's System of Therapeutics. The character of the scientific work done under his direction is well illustrated in the first volume of Medical Reports issued by the hospital in 1903, and containing articles by Paton, Farrar, Dunton and Rusk, all of them at the present time leaders of psychiatric research. Dr. Brush early recognised the opportunities at Sheppard for affording training to young men in psychiatry. In his fourth annual report he says: "As we are able to add to our means of research and to accumulate here a library of reference and gather material for study in the way of clinical, laboratory and pathological notes, we shall attract to the work those who will not only aid in the distinctively medical work of the institution, but will go from here to extend its work in other fields."

Dr. Brush is of the opinion that an air of comfort or even luxury about the hospital has a distinctly curative influence upon such patients. In Hare's Therapeutics, above quoted, he holds that among the special features of hospital care should be mentioned the influence of the institution—the effect of cheerful surroundings, of decorated walls, bright rooms, a pleasant outlook, flowers, books and pictures; in short, what may be termed the esthetic of the patient's environment are all for his good. "I have seen," says Dr. Brush, "the entire character of a ward changed, so far as the conduct of the patients was concerned, by the addition of a large sitting-room, the frescoing of the walls and the instruction of homelike furniture, pictures and books."

In 1882 Dr. Brush visited a number of the asylums and hospitals for the insane of Great Britain, carefully noting details of value for his own work. A report of his visit was made in the *American Journal of Insanity* for January, 1883, and the *Bulletin de la Société de Medécine Mentale de Belgique.* In 1902 he visited some of the best psychiatric clinics of Germany and hospitals of France and Great Britain. A careful analysis of the methods pursued in the German clinics and a comparison of these with our American methods was presented by him in the *American Journal of Insanity* in 1905, and reprinted. He also made visits to Europe with the purpose of studying hospital methods in 1890 and 1896. Last year he went to Amsterdam as a delegate to the International Congress of Psychiatry and Neurology, and took advantage of the opportunity to visit some of the psychiatric hospitals of France and England.

Dr. Brush has been connected with medical journalism during almost his entire professional career. From 1874 to 1879 he was editor of the *Buffalo Medical and Surgical Journal,* and while at the State Hospital at Utica was on the staff of the *American Journal of Insanity,* one of the oldest special medical periodicals in the world. Since 1897 he has again been one of the editors of the *Journal of Insanity,* now the official organ of the American Medico-Psychological Association, and is now its managing editor. Besides being a member of various American medical societies, he is an honorary member of the Medico-Psychological Association of Great Britain and Ireland and of the Sociéte de Médécine Mentale of Belgium, and foreign associate member of the Societé Médico-Psychologique of Paris.

